# Assessment of heart rate deceleration capacity, heart rate deceleration runs, heart rate acceleration capacity, and lipoprotein-related phospholipase A2 as predictors in individuals with dementia

**DOI:** 10.3389/fneur.2024.1438736

**Published:** 2025-01-09

**Authors:** Yaping Wang, Mingyan Liu, Yaping Liu, Xiaowei Tang, Xiangming Tang

**Affiliations:** ^1^Department of Electrocardiogram, Yangzhou Wutaishan Hospital of Jiangsu Province, Teaching Hospital of Yangzhou University, Yangzhou, China; ^2^Department of Clinical Laboratory, Yangzhou Wutaishan Hospital of Jiangsu Province, Teaching Hospital of Yangzhou University, Yangzhou, China; ^3^Department of Psychiatry, Yangzhou Wutaishan Hospital of Jiangsu Province, Teaching Hospital of Yangzhou University, Yangzhou, China; ^4^Department of Neurology, The Affiliated Taizhou People’s Hospital of Nanjing Medical University, Taizhou School of Clinical Medicine, Nanjing Medical University, Taizhou, Jiangsu, China

**Keywords:** vascular dementia, Alzheimer’s disease, heart rate deceleration capacity, heart rate deceleration runs, heart rate acceleration capacity, autonomic dysfunction, serum lipoprotein-related phospholipase A2

## Abstract

**Background:**

Autonomic dysfunction plays an essential role in dementia, however, it is not known whether electrocardiogram autonomic dysfunction-related indicators are associated with the severity of dementia. In this study, we attempted to investigate whether these indicators are correlated in patients with vascular dementia and Alzheimer’s disease compared with normal health individuals. For this purpose, we measured and analyzed the predictive value of heart rate deceleration capacity (DC), heart rate deceleration runs (DRs), heart rate acceleration capacity (AC) along with the plasma levels of lipoprotein-associated phospholipase A2 (Lp-PLA2).

**Methods:**

We compared 83 dementia cases including 41 vascular dementia (VD), 42 Alzheimer’s disease (AD) patients with 42 elderly health controls. The Mini-Mental State Examination (MMSE) scores, DC, DRs, AC, and Lp-PLA2 levels were comprehensively evaluated.

**Results:**

Our studies showed that DC and DRs in VD and AD groups were significantly lower than those in controls, while AC values were significantly higher. Furthermore, the risk stratification (high- and moderate-) of DC, DRs, and AC in VD and AD groups was increased, while the low-risk was simultaneously decreased. In addition, DC and DRs were positively while AC and Lp-PLA2 were negatively correlated with MMSE scores. Logistic regression analysis indicated that DC, DRs, AC, and Lp-PLA2 were associated with dementia. Moreover, the areas under the ROC curves showed that the combination of five variables and AC + Lp-PLA2 were 0.970 (95% CI, 0.923–0.992) and 0.940 (95% CI, 0.882–0.974) were larger than each single indicator alone.

**Conclusion:**

Distinctive alterations in dynamic electrocardiogram-related indicators reveal a decline in autonomic nervous functions among individuals with dementia. By incorporating comprehensive analyses of DC, DRs, AC, and Lp-PLA2 values, the specificity and sensitivity of dementia diagnosis can be significantly enhanced.

## 1 Introduction

Dementia is a severe neurocognitive disorder characterized by a chronic progressive loss of cognitive function. Many factors are involved in the development of dementia, such as genetic factors, environmental factors, and aging. Currently, there is no effective treatment for dementia and supportive therapies are available for the management of this disorder. There are various forms of dementia, with Alzheimer’s disease (AD) and vascular dementia (VD) being the two most prevalent types (1). As the population ages, the incidence of AD and VD is on the rise. AD typically manifests with a gradual onset and early symptoms primarily involving memory loss, which is attributed to neurotoxicity resulting from abnormal deposits of beta-amyloid in the brain and excessive phosphorylation of tau proteins (2). In contrast, VD is a clinical syndrome characterized by cognitive and intellectual impairment, resulting from brain injuries such as hemorrhage, ischemic stroke, small vessel disease, white matter damage, and neuronal injury (1). However, there is growing evidence that AD and VD share similar risk factors and that there is some overlap and commonality in their pathological mechanisms (3). For instance, beta-amyloid deposition and decreased clearance ability are also observed in the brains of VD patients (3). Despite some progress in dementia research, the complex pathological mechanisms and the progressive nature of the disease in most patients underscore the importance of finding biological markers that reflect the severity of dementia for assessing the condition and following the disease.

Studies demonstrated that patients with early dementia, even before cognitive dysfunction, have autonomic dysfunction, which gradually worsens with the progression of disease (4, 5). The clinical manifestations of dementia involve multiple aspects of the autonomic nervous system (6), including postural hypotension, swallowing disorders, and urination disorders (7–9), as well as constipation, abnormal sweating, sexual dysfunction, and sleep disorders (10–13). Studies suggested that the development of autonomic dysfunction in dementia is mainly associated with neuroanatomic damage and neurochemical changes in the central autonomic nervous network (14). The central autonomic nervous system governs autonomic function and plays a crucial role in regulating cognitive, behavioral, and endocrine responses. Subcortical structures such as the amygdala, lateral nucleus, hypothalamus, striatum, thalamus, brain stem, and others are likely involved in the regulation of neurodegenerative diseases’ pathogenesis, particularly through their interactions with autonomic nerves. It has been demonstrated that amygdala volume was significantly reduced in AD patients, with a loss of up to 50% of neurons (14). In the progression of dementia, neurochemical changes occur, particularly affecting the cholinergic system. These changes are characterized by increased acetylcholinesterase activity in the insula, decreased cortical acetylcholine transferase activity, and impaired cholinergic synaptic transmission. Consequently, these alterations contribute to autonomic dysfunction (15). Autonomic dysfunction has been observed in both AD and VD patients, as reported by several studies (16, 17), suggesting that AD and VD patients share common features in the magnifications of autonomic dysfunction.

In addition, many studies demonstrated that vascular inflammatory reactions are involved in the development of dementia. The vascular inflammatory markers were elevated in patients with dementia (18), such as lipoprotein-associated phospholipase A2 (Lp-PLA2), which is a novel vascular inflammatory marker that mediates vascular inflammatory reactions by regulating lipid metabolism (19). The latest clinical study suggested that Lp-PLA2 is closely associated with cognitive impairment and is expected to be used as a hematological risk marker for evaluating the onset of AD and VD (20). In patients with dementia, the treatment of Lp-PLA2 inhibitors can prevent the disease progression and alleviate the symptoms of dementia (18, 21). Studies showed that Lp-PLA2 inhibition can improve executive functions and working memory in patients with dementia when examining the effects of Lp-PLA2 inhibition on cognitive function in AD and VD patients (20). Specifically, the Lp-PLA2 inhibitor, darapladib, has been shown to improve these cognitive functions (4). Additionally, mechanistic studies indicated that Lp-PLA2 inhibitors can alleviate damage to the blood–brain barrier and reduce the aggregation of A*β*1-42 in the brain, suggesting that the protective effect of Lp-PLA2 inhibitors in dementia may be related to the clearance of brain β-amyloid proteins associated with the blood–brain barrier (4). Although some other studies failed to demonstrate a significant correlation between serum Lp-PLA2 levels and the development of dementia (22).

Heart rate deceleration capacity (DC), heart rate deceleration runs (DRs), and heart rate acceleration capacity (AC) are non-invasive indicators of the function of the autonomic nervous system in the heart. These indicators are currently widely used in clinical examination of cardiac electrical activity and can be systematically and automatically calculated, making them convenient and fast for assisting in the assessment of disease prognosis and severity (23). However, the clinical value of these indicators in the risk assessment of dementia patients remains uncertain. Therefore, to explore the predictive value of these autonomic nervous system-related markers in the severity of dementia, we evaluated the characteristics of DC, DRs, and AC in dynamic electrocardiogram (DCG) along with the serum inflammatory marker Lp-PLA2 levels in dementia patients. Our studies will not only increase the understanding of the relationship between dementia and the autonomic nervous system but also provide new perspectives and strategies for the prevention and treatment of dementia.

## 2 Materials and methods

### 2.1 Study subjects

Eighty-three newly diagnosed dementia patients from October 2022 to October 2023 were included as subjects for this study, including 41 cases in the vascular dementia group (VD group) (24 males and 17 females) and 42 cases in the Alzheimer’s disease group (AD group) (23 males and 19 females). Furthermore, a control group consisting of 42 elderly healthy individuals (20 males and 22 females) who underwent physical examinations during the same period was selected. All participants were Han Chinese. The diagnostic criteria for the patients included in our study are as follows: for AD diagnosis, we referred to the criteria established by the National Institute on Aging and the Alzheimer’s Association in 2011 (24). For VD diagnosis, we referred to the criteria set by the National Institute of Neurological Disorders and Stroke and the Association Internationale pour la Recherche et l’Enseignement en Neurosciences (NINDS-AIREN) (25). Exclusion criteria: (1) After pacemaker implantation, (2) Patients complicated with atrial fibrillation and atrial flutter, (3) Patients with more than two degrees of atrioventricular block, (4) Myocardial infarction and cardiomyopathy, (5) Patients complicated with hyperthyroidism or hypothyroidism, (6) Diabetes, liver and kidney dysfunction, (7) Electrolyte disorders, (8) Severe physical diseases, organic brain diseases, or mental disorders that affect cognitive functions, (9) Patients who taken antiarrhythmic drugs or other drugs that affect autonomic nervous function within 2 weeks (24, 25). The control group consisted of elderly individuals who were age and gender-matched and participated in routine health check-ups. The cognitive functions of the control group were normal, characterized by the absence of any abnormalities on brain CT or MRI scans, and the absence of neurological or psychiatric conditions that could potentially affect cognitive functions. We first clarified the purpose of the study and the requirements for participants, then published recruitment information through appropriate channels, which included study details, participant eligibility, commitments, and incentives. We ensured the process was transparent, and honest, and that participant privacy was protected. This study was approved by the hospital ethics committee (WTSLL2022010) and registered in the Nation Medical Research Registry System (MR-32-24-051272). All enrolled subjects provided informed consent.

### 2.2 Methods

#### 2.2.1 Dynamic electrocardiogram examination

A 12-lead dynamic electrocardiogram (Holter) recorder was used to continuously collect 24-h ECG signals from all subjects, which were imported into the DMS300-4Asystem. After the manual removal of interference, the DC, DRs, and AC values were obtained and calculated by a computer via the method of phase rectified signal averaging (PRSA) (23, 26). The DRs mainly used DR2, DR4, and DR8 values. Risk stratification was performed post-diagnosis based on DC, AC, and DRs values: DC value ≤ 2.5 ms was high-risk, DC value 2.6–4.5 ms was moderate-risk, and DC value >4.5 ms was low-risk; the absolute value of AC risk stratification was the same as DC value; DR4 ≤ 0.05% was high-risk; DR4 > 0.05%, but DR2 ≤ 5.4% or DR8 ≤ 0.005% was moderate-risk; DR4 > 0.05% and DR2 > 5.4% and DR8 > 0.005% was low-risk (27).

#### 2.2.2 MMSE score

The MMSE includes six aspects: orientation, memory, attention, calculation, recall ability, and language ability. The total score is 30, the higher MMSE score indicates better cognitive function. In our study, dementia patients were included based on their education level and scores as follows: illiterate patients (MMSE≤17), patients with an elementary school education (MMSE≤20), and patients with a junior high school education or above (MMSE≤26).

#### 2.2.3 Lp-PLA2 detection

Five milliliters of peripheral venous blood was collected from all subjects on a fasting state in the morning, placed in a vacuum collection vessel containing EDTA, and centrifuged at 3000 r/min for 10 min using an ultracentrifuge. The supernatant was isolated and stored at −80°C. The serum Lp-PLA2 levels were measured by using the method of enzyme-linked immunosorbent assay (ELISA), which was strictly performed according to the procedures of the ELISA kit (EH304RB, Thermo Fisher Scientific, MA, United States).

#### 2.2.4 Statistical analysis

Data were analyzed using SPSS version 25.0 software. Each variable was examined for normal distribution. Continuous variables with normal distribution were expressed as mean ± SD. Analysis of variance was used for comparison between multiple groups, and the least significant difference test was used for further comparison between two groups. Continuous variables with a skewed distribution were expressed as a median and interquartile range, and a rank sum test was used. The categorical variables were expressed as frequency and percentage and were made using the chi-square test or Fisher’s exact test. Correlations between autonomic parameters and MMSE scores were calculated using the Spearman correlation test. Logistic regression analysis was applied to determine independent factors associated with dementia in patients. MedCalc version 22.0 software was used to draw the receiver operating characteristic (ROC) curve to analyze the diagnostic efficacy of each indicator. A value of *p* < 0.05 was considered statistically significant.

## 3 Results

Firstly, we examined the baseline characteristics of dementia and the control group. As shown in Table 1, compared with the control group, the MMSE scores in the VD group and AD group were significantly lower (*p* < 0.05), while the Lp-PLA2 levels were significantly higher (*p* < 0.05). There were no significant differences in gender, age, BMI, blood pressure, HDL-C, LDL-C, TG, and TC among the three groups (*p* > 0.05, Table 1).

**Table 1:** Comparison of the baseline characteristics in each group.

Feature	VD (*n* = 41)	AD (*n* = 42)	Control (*n* = 42)	*F*/Z/*χ*^2^-value	*p*-value
Male/female (*n*)	24/17	23/19	20/22	1.029	0.598
Age (year)	70.76 ± 7.00	71.07 ± 4.38	68.29 ± 6.95	2.51	0.085
BMI (kg/m^2^)	24.43 ± 1.80	23.67 ± 1.74	23.81 ± 2.13	1.868	0.159
Duration (year)	5(3,8)	6(4,11)	–	−1.595	0.111
SBP (mm/Hg)	129.27 ± 8.80	128.43 ± 10.51	123.57 ± 5.48	3.777	0.056
DBP (mm/Hg)	82.12 ± 8.29	79.31 ± 6.04	80.38 ± 4.53	3.194	0.054
FPG (mmol/L)	5.10 ± 0.58	5.05 ± 0.57	4.95 ± 0.53	0.784	0.459
HDL-C (mmol/L)	1.06 ± 0.28	1.08 ± 0.25	1.17 ± 0.26	2.165	0.119
LDL-C (mmol/L)	2.45 ± 0.51	2.30 ± 0.52	2.38 ± 0.40	1.76	0.177
TG (mmol/L)	1.41 ± 0.58	1.17 ± 0.48	1.53 ± 0.76	1.307	0.274
TC (mmol/L)	4.33 ± 0.99	4.20 ± 0.96	4.65 ± 0.94	2.671	0.073
MMSE scores	12.73 ± 3.27	15.26 ± 3.27	25.31 ± 2.33	206.79	<0.0001
Lp-PLA2 (μg/L)	201.02 ± 20.60	184.84 ± 12.79	136.36 ± 9.68	239.73	<0.0001

Data were presented as mean ± SD or medium. BMI, body mass index; SBP, systolic blood pressure; DBP, diastolic blood pressure; FPG, fasting blood glucose; HDL-C, high-density lipoprotein cholesterol; LDL-C, low-density lipoprotein cholesterol; TG, triglyceride; TC, total cholesterol; MMSE, mini-mental state examination; Lp-PLA2, lipoprotein-associated phospholipase A2.

To investigate whether DC, DRs, and AC can be used as indicators of autonomic dysfunction in dementia patients, we analyzed the features of these variables in different groups. As shown in Table 2, DC, DRs, and AC in the VD group and AD group showed prominent changes. Compared with the control group, DC and DRs in the VD group and AD group were significantly lower (*p* < 0.05), while AC in the VD group and AD group was significantly higher than that in the control group (*p* < 0.05). However, compared with the VD group, DC, DRs, and AC values in the AD group showed no significant changes (*p* > 0.05, Table 2). These findings suggested that patients with VD or AD exhibited characteristic dynamic electrocardiogram changes of characteristics of autonomic dysfunction, indicating that these patients had autonomic dysfunction.

**Table 2:** Comparison of DC, DRs, and AC in each group.

Parameters	VD (*n* = 41)	AD (*n* = 42)	Control (*n* = 42)	*F*/*Z* value	*P*-value
DC (ms)	3.15(2.24,4.00)	3.58(2.45,4.54)	6.71(5.45,7.89)	45.916	<0.0001
DR2 (%)	6.11 ± 1.85	6.57 ± 2.69	8.20 ± 2.07	6.507	0.002
DR4 (%)	0.14(0.09,0.24)	0.11(0.07,0.26)	0.7(0.4,1.0)	53.809	<0.0001
DR8 (%)	0.01(0.00,0.05)	0.00(0.00,0.01)	0.02(0.01,0.03)	55.378	<0.0001
AC (ms)	−3.36(−4.20,−2.39)	−4.1(−5.43,−3.03)	−7.0(−8.50,−5.70)	47.437	<0.0001

Data were presented as mean ± SD or medium. DC, heart rate deceleration capacity; DRs, heart rate deceleration runs; AC, heart rate acceleration capacity.

Furthermore, to explore the significance of changes in ambulatory electrocardiograms in the severity of dementia, we analyzed the significance of different risk levels of DC, DRs, and AC in dementia patients. As shown in Table 3, there were significant differences in the risk stratification of DC, DRs, and AC among the VD group, AD group, and control group (*p* < 0.05). The high- and moderate-risk stratification of DC, DRs, and AC in the VD group and AD group were significantly higher than that in the control group (*p* < 0.05). However, the low-risk stratification of DC, DRs, and AC in the VD group and AD group was significantly lower than that in the control group (*p* < 0.05). These results indicated that DC, DRs, and AC probably can be used as indicators for risk stratification in dementia patients.

**Table 3:** Comparison of risk stratification of DC, DRs, and AC in each group.

Parameters		VD (*n* = 41)	AD (*n* = 42)	Control (*n* = 42)	*χ*^2^-value	*P*-value
DC	Low-risk	7(17.08)	9(21.42)	37(88.10)	19.882	<0.0001
	Moderate-risk	15(36.59)	7(16.67)	4(9.5)	6.236	0.044
	High-risk	19(46.34)	26(61.90)	1(2.3)	22.546	<0.0001
DRs	Low-risk	9(21.95)	1(2.38)	40(95.24)	33.662	<0.0001
	Moderate-risk	7(17.01)	12(28.58)	2(4.76)	6.154	0.046
	High-risk	25(60.98)	29(69.05)	0(0)	31.826	<0.0001
AC	Low-risk	3(7.32)	14(33.33)	38(90.48)	23.323	<0.0001
	Moderate-risk	22(53.66)	18(42.86)	3(7.14)	11.954	0.003
	High-risk	16(39.02)	10(23.81)	1(2.38)	11.237	0.004

Data were presented as count (%). DC, heart rate deceleration capacity; DRs, heart rate deceleration runs; AC, heart rate acceleration capacity.

The MMSE score is widely used as a tool to evaluate the severity of dementia in patients. We found that DC, DR2, DR4, and DR8 in patients with dementia were positively correlated with MMSE scores (*r* = 0.952, *r* = 0.977, *r* = 0.849, *r* = 0.403, *p* < 0.05), while AC and Lp-PLA2 were negatively correlated with MMSE scores (*r* = −0.901, *r* = −0.964, *p* < 0.05). These results showed that DC, DRs, AC, and Lp-PLA2-related indicators are associated with the MMSE scores, suggesting these variables can reflect the severity of dementia. In addition, as shown in Table 4, we investigated these factors in dementia patients using logistic regression analysis and found that DC, DRs, AC, and Lp-PLA2 were all factors associated with dementia patients (*p* < 0.05).

**Table 4:** Logistic regression analysis of factors associated with dementia in patients.

Parameters	*B*	BE	Wald *χ*^2^-value	OR (95%CI)	*P*-value
DC	−0.517	0.109	22.288	0.596(0.481 ~ 0.739)	<0.0001
DR2	−0.294	0.096	9.427	0.745(0.618 ~ 0.899)	0.002
DR4	−6.271	1.144	30.077	0.002(0.000 ~ 0.018)	<0.0001
DR8	−0.246	21.666	16.215	0.034(1.532 ~ 1.675)	<0.0001
AC	0.510	0.102	25.167	1.665(1.364 ~ 2.032)	<0.0001
Lp-PLA2	0.271	0.081	11.181	1.312(1.119 ~ 1.538)	0.001

DC, heart rate deceleration capacity; DRs, heart rate deceleration runs; AC, heart rate acceleration capacity; Lp-PLA2, lipoprotein-associated phospholipase A2.

**Figure 1:**
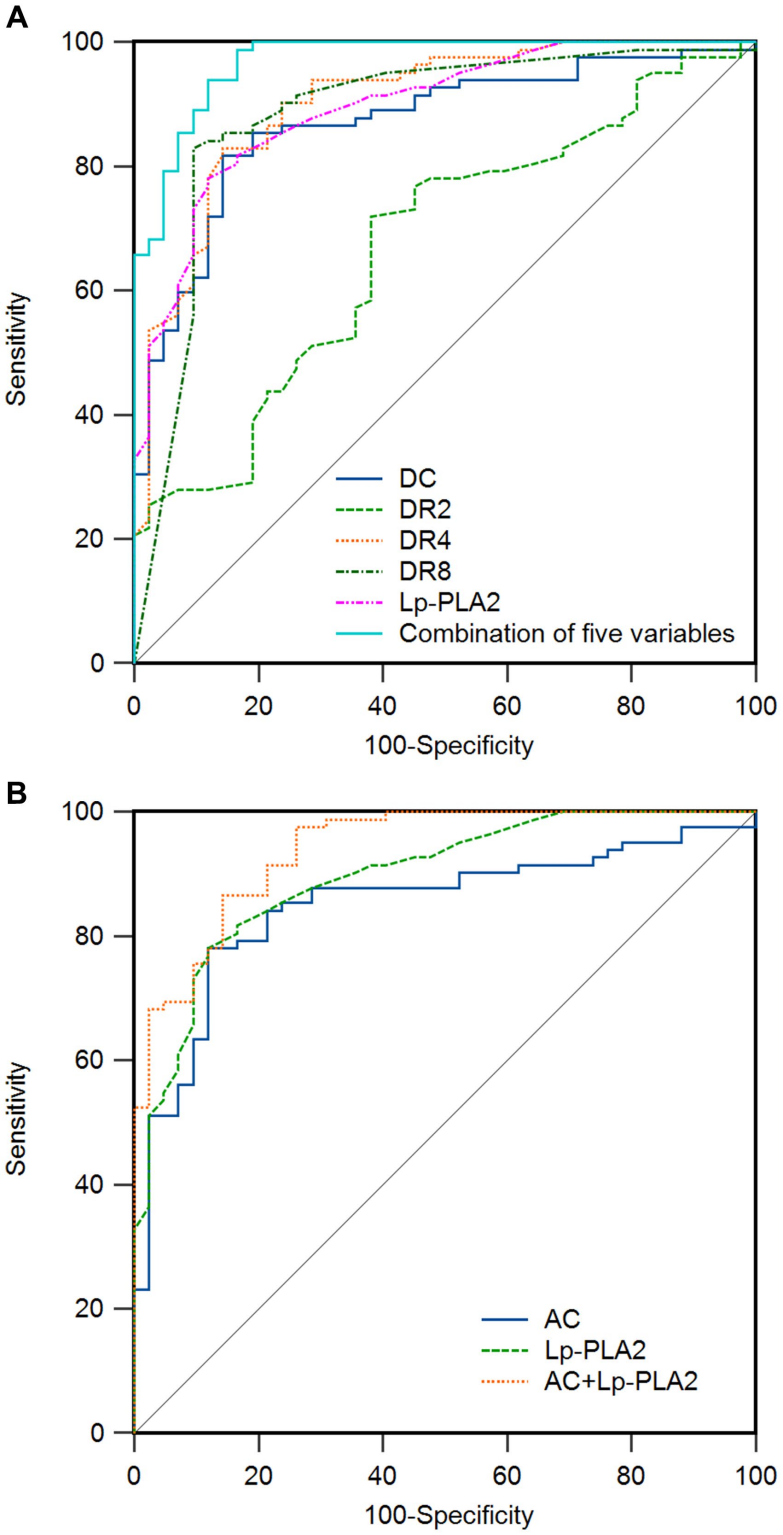
Receiver operating characteristic (ROC) curve of related indicators for the prediction of dementia in patients. **(A)** The area under the curve (AUC) of deceleration capacity (DC), heart rate deceleration 2 (DR2), heart rate deceleration 4 (DR4), heart rate deceleration 8 (DR8), lipoprotein-associated phospholipase A2 (Lp-PLA2), and combination of five variables for the prediction of dementia are 0.870, 0.673, 0.903, 0.884, 0.896, and 0.970, respectively. **(B)** The AUC of heart rate acceleration capacity (AC), Lp-PLA2, and AC + Lp-PLA2 for the prediction of dementia are 0.849, 0.896, and 0.940. The ideal AUC is 1.0. The reference line (black, diagonal line) represents an AUC of 0.5, which is based on chance alone.

Moreover, to further examine the predictive value of DC, DRs, AC, and Lp-PLA2 for dementia, we analyzed the ROC curve of each indicator. As shown in Figure 1 and Table 5, the ROC curve results showed that each indicator had a good predictive value for dementia. The areas under the ROC curves for DC, DR2, DR4, DR8, Lp-PLA2, and combination of five variables diagnosis were 0.870 (95%CI:0.798 ~ 0.924, *p* < 0.01), 0.673 (95%CI:0.583 ~ 0.754, *p* < 0.01), 0.903 (95%CI:0.836 ~ 0.949, *p* < 0.01), 0.884 (95%CI:0.814 ~ 0.935, *p* < 0.01), 0.896 (95%CI:0.828 ~ 0.943, *p* < 0.01) and 0.970 (95%CI:0.923 ~ 0.992, *p* < 0.01), respectively (Figure 1A). In addition, the combination of five variables diagnosis had a significantly larger area under the ROC curve than DC, DR2, DR4, DR8, and Lp-PLA2 (*Z* = 3.464, *Z* = 5.952, *Z* = 2.517, *Z* = 2.558, *Z* = 3.056, *p* < 0.01). As shown in Figure 1B, the areas under the ROC curves for AC and AC + Lp-PLA2 were 0.849 (95%CI: 0.774 ~ 0.907, *p* < 0.01) and 0.940 (95% CI: 0.882–0.974, *p* < 0.01), respectively, indicating that the combined diagnosis of AC + Lp-PLA2 exhibited a significantly larger area under the ROC curve than AC (*Z* = 2.908, *p* < 0.01). Given the observed sex differences in the prognosis of AD, we aimed to evaluate the diagnostic capabilities of these biomarkers between male and female AD patients. As shown in Table 6, the comparisons of DC, DR2, DR4, DR8, AC, and Lp-PLA2 between male and female AD patients exhibited no significant differences. Furthermore, the areas under the ROC curves for AC and AC + Lp-PLA2 were 0.746 and 0.957, respectively, for DC, DR2, DR4, DR8, Lp-PLA2, and the combined five-variable diagnosis were 0.772, 0.891, 0.872, 0.835, 0.963, and 0.972, respectively, for male AD patients (Supplementary Figures S1A,B). In female AD patients, the areas under the ROC curves for AC and AC + Lp-PLA2 were 0.764 and 0.925, respectively, and for DC, DR2, DR4, DR8, Lp-PLA2, and the combined five-variable diagnosis were 0.810, 0.665, 0.876, 0.897, 0.852, and 0.985, respectively (Supplementary Figures S1C,D), suggesting that these biomarkers did not show sex difference in diagnostic capabilities in the current study.

**Table 5:** Analysis of the value of related indicators in predicting dementia.

Parameters	Sensitivity	Specificity	Truncation value	AUC	95%CI	*P*-value
DC	81.71	85.71	4.7634	0.870	0.798 ~ 0.924	<0.0001
DR2	71.95	61.9	7.7	0.673	0.583 ~ 0.754	<0.0001
DR4	82.93	85.71	0.27	0.903	0.836 ~ 0.949	<0.0001
DR8	82.93	90.48	0.0043	0.884	0.814 ~ 0.935	<0.0001
Lp-PLA2	78.05	88.10	160	0.896	0.828 ~ 0.943	<0.0001
Combination of five variables	98.78	83.33	–	0.970	0.923 ~ 0.992	<0.0001
AC	78.05	88.10	5.1059	0.849	0.774 ~ 0.907	<0.0001
AC + Lp-PLA2	86.85	85.71	–	0.940	0.882 ~ 0.974	<0.0001

DC, heart rate deceleration capacity; DRs, heart rate deceleration runs; AC, heart rate acceleration capacity; Lp-PLA2, lipoprotein-associated phospholipase A2.

**Table 6:** Comparison of DC, DRs, and AC between male and female AD patients.

Parameters	Male AD (*n* = 23)	Female AD (*n* = 19)	*t*/*Z* value	*P*-value
DC (ms)	3.38 ± 1.09	3.53 ± 0.68	−0.097	0.923
DR2 (%)	5.99 ± 2.03	6.28 ± 3.23	−1.602	0.117
DR4 (%)	0.22 ± 0.09	0.18 ± 0.07	1.157	0.254
DR8 (%)	0.00(0.00,0.00)	0.00(0.00,0.00)	−0.829	0.436
AC (ms)	−4.41 ± 1.10	−5.1 ± 1.16	0.870	0.390
LP-LPA2 (μg/L)	192.17 ± 12.98	186.21 ± 7.62	1.622	0.113

Data were presented as mean ± SD or medium. DC, heart rate deceleration capacity; DRs, heart rate deceleration runs; AC, heart rate acceleration capacity.

In addition, the optimal cut-off values for predicting VD with DC, DR2, DR4, DR8, AC, and Lp-PLA2 were 4.5557 ms, 7.7, 0.27, 0.0061%, −5.1059 ms, and 167 μg/L, respectively (Table 7). Moreover, the optimal cut-off values for predicting AD with DC, DR2, DR4, DR8, AC, and Lp-PLA2 were 4.9919 ms, 8.1, 0.45, 0.0036%, −5.65 ms, and 158 μg/L, respectively.

**Table 7:** Predictive value of relevant indicators for VD and AD.

Parameters	VD		AD	
	AUC	Truncation value	AUC	Truncation value
DC	0.893	4.5557 ms	0.828	4.9919 ms
DR2	0.648	7.7%	0.711	8.1%
DR4	0.97	0.27%	0.886	0.45%
DR8	0.846	0.0061%	0.906	0.0036%
AC	0.92	−5.1059 ms	0.787	−5.65 ms
Lp-PLA2	0.927	167 μg/L	0.993	158 μg/L

DC, heart rate deceleration capacity; DRs, heart rate deceleration runs; AC, heart rate acceleration capacity; Lp-PLA2, lipoprotein-associated phospholipase.

## 4 Discussion

Dementia develops as a result of the damage and degeneration of brain nerve cells, disrupting the communication between neurons and ultimately causing a decline in cognitive function. The impairment of cognitive function can lead to a gradual decline in an individual’s functional abilities in daily life, and affect their social, emotional, and behavioral performance (28). It is well known that dementia is not a normal aging process, but a condition caused by many factors. Approximately 50 million people worldwide suffer from dementia, and it is expected to increase to 152 million by 2050, especially in low-income and middle-income countries (29). Dementia not only has a serious impact on individual patients but also imposes a significant burden on families and society, with an estimated annual cost of approximately $1 trillion worldwide (29). It has been demonstrated that many factors can increase the risk of dementia, including midlife hypertension and obesity, hearing loss, traumatic brain injury, alcohol abuse, smoking, depression, inactivity, social isolation, and diabetes (28). It is widely believed that the most common causes of dementia are AD and VD. AD is mainly caused by abnormal aggregation of two types of proteins, *β*-amyloid protein and Tau protein (30). *β-*amyloid accumulation leads to the formation of extracellular neuronal plaques leading to neurodegeneration. The abnormal aggregation of hyperphosphorylated tau proteins leads to a collapse of axonal microtubules and the subsequent development of neurofibrillary tangles. Both pathologies lead to neuronal cell death and brain atrophy. The mechanism of VD is associated with cerebrovascular diseases, where stroke (cerebrovascular events) or multiple small cerebrovascular lesions lead to ischemia and hypoxia in the brain, damaging nerve cells, and brain tissue. These lesions may lead to cognitive decline and dementia symptoms (31).

Lp-PLA2 is a class of enzymes that can catalyze the hydrolysis of phospholipid substances such as phosphatidylcholine, degenerating them into free fatty acids and lysozyme A2 (32). Lp-PLA2 has a variety of physiological functions, including maintaining the balance of lipid metabolism, repairing and maintaining the cell membrane, and regulating inflammatory responses. However, the abnormally high level of activity of Lp-PLA2 may be associated with the occurrence and development of a variety of diseases, including cardiovascular diseases, inflammatory diseases, and nervous system diseases. In nervous system diseases, it has been found that Lp-PLA2 is an independent risk factor for cognitive impairment in patients with dementia (33). The increased activity of Lp-PLA2 leads to the release of phosphatidylcholine in brain tissue, which leads to the production of excess fatty acids, oxidative stress, and neuroinflammatory responses, resulting in the decline of cognitive function and the occurrence of dementia symptoms. In this study, the Lp-PLA2 level in the VD group and the AD group was significantly higher than that in the control group, and the Lp-PLA2 level was negatively correlated with the MMSE score of dementia patients, indicating that the higher the Lp-PLA2 level, the more serious the cognitive impairment of patients, which is consistent with the viewpoint of Maher-Edwards et al. (20). They found that the plasma Lp-PLA2 level in AD patients was significantly increased, and the use of Lp-PLA2 inhibitors can reduce the deposition of *β*-amyloid protein, thereby alleviating the symptoms of AD patients.

The central nervous system regulates the function of the heart and blood vessels through the two branches of the autonomic nervous system, sympathetic nerve and parasympathetic nerve. AC mainly refers to the ability of the cardio accelator nerves of the sympathetic nervous system to increase heart rate (34). Under normal conditions, when the heart rate needs to be increased, the sympathetic nerve will be activated, resulting in an increased heart rate to cope with stress or exercise and other activities, shown as a decreased AC value. DC refers to the ability of parasympathetic nerve activity to affect heart rate (35). It mainly regulates heart rate through the vagus nerve, which will decrease the heart rate under normal conditions to maintain the stability of the heart, shown as an increased DC value. DRs refer to the ability of the heart to be regulated by the vagus nerve in a short time, which complements DC and can improve the accuracy of cardiac risk stratification (27). The damage of nerve cells in patients with dementia will affect the regulating function of the autonomic nervous system, resulting in the impact of DC, DRs, and AC (36). This study found that DC and DRs in the VD and AD groups were significantly lower than those in the control group, and AC was significantly higher than those in the control group. The risk stratification of intermediate and high-risk strata was significantly higher than that of the control group, while the low-risk strata was significantly lower than that of the control group. Correlation analysis showed that DC, DRs, and MMSE scores in patients with dementia were positively correlated, and AC was negatively correlated with MMSE scores. DC and DRs reflected the function of the vagus nerve, and AC reflected the function of the sympathetic nerve, indicating that the autonomic nervous function regulation of patients with dementia was abnormal, which may further affect the cardiovascular health of patients and even cause cardiovascular events. Logistic regression analysis showed that DC, DRs, AC, and Lp-PLA2 were all influencing factors in patients with dementia. The ROC curve results showed that DC, DRs, AC, and Lp-PLA2 had good predictive value for dementia. Furthermore, this study found that the area under the curve of DC, DRs, and AC combined with Lp-PLA2 was 0.970 (95%CI: 0.923–0.992) and 0.940 (95%CI: 0.882–0.974), indicating that DC, DRs, AC combined with Lp-PLA2 had higher diagnostic value for dementia. The decrease in DC may be one of the early indicators of cognitive impairment. Researchers used dynamic electrocardiograms and cognitive assessment tools to track a group of elderly people for a long time and found that in the early stage of cognitive decline, DC had been significantly reduced (37). This suggests that DC can help identify potential cognitive impairment. This study found that patients with dementia had autonomic nervous function impairment, which is consistent with the report in the literature (38). It can be seen that DC, and AC combined with Lp-PLA2 is more accurate and comprehensive for the screening of dementia patients. This study also found that the best cut-off values of DC, DR2, DR4, DR8, AC and Lp-PLA2 for predicting VD were 4.5557 ms, 7.7, 0.27, 0.0061%, −5.1059 ms and 167 μg/L, respectively, and the best cut-off values of DC, DR2, DR4, DR8, AC and Lp-PLA2 for predicting AD were 4.9929 ms, 8.1, 0.45, 0.0036%, −5.65 ms and 158 μg/L, respectively. This also provides certain reference values for clinical practice. Therefore, clinical attention should be paid to the regular detection and evaluation of DC, DRs, AC, and Lp-PLA2 in patients with dementia. The findings indicate that DC, DRs, AC, and Lp-PLA2 may serve as potential biological biomarkers for the evaluation of dementia, particularly in both early-stage and late-stage patients, acting as a supplementary strategy. However, they cannot replace the commonly used scales, such as the MMSE, in the assessment of dementia. Therefore, monitoring and intervening in the function of autonomic nerves helps maintain the heart health of patients and improve their living conditions. Although previous studies indicated that the progression of AD exhibits a gender-related pattern, with female patients typically showing more pronounced hippocampal atrophy, higher amyloid burden, and a more rapid clinical decline following a dementia diagnosis (39), this study did not find these biomarkers to show sex difference in diagnostic capabilities. This may be attributed to the small sample size and the severity of dementia among participants.

The current study suggests that the ECG/Lp-PLA2 biomarker panel holds promise as a clinical biomarker. As previously mentioned, dynamic electrocardiography is widely employed in clinical settings, offering the advantage of automatically calculating and acquiring relevant indicators, which is both convenient and rapid, making them suitable for disease diagnosis and assessment. The detection of serum inflammatory factor levels is relatively straightforward, characterized by objectivity, cost-effectiveness, and ease of operation. By combining these two types of biological markers, it is possible to mitigate, to some extent, the practical issues associated with the subjectivity and low sensitivity of scale assessments, as well as the significant economic burden and poor patient tolerance of imaging examinations. Therefore, they have the potential to serve as auxiliary biological markers for the diagnosis of dementia. Despite this, there are some limitations in the current study. Firstly, this study is a preliminary cross-section study with a relatively small size of samples, and selection bias may affect the results. Secondly, the study only included patients with sinus rhythm, and the results do not apply to patients with non-sinus rhythm such as atrial fibrillation or atrial flutter. In addition, the current study did not incorporate serum Aβ and p-tau, which are well-established biomarkers for the evaluation of AD patients, partly because the diagnosis of VD primarily relies on clinical and imaging findings, as there is a lack of universally recognized biomarkers for VD. Therefore, the results need to be further confirmed by larger-scale and further studies.

In conclusion, characteristic changes in DC, DRs, and AC can reflect the severity of autonomic dysfunction in dementia patients. In combination with Lp-PLA2, it can improve the assessment and clinical prediction value of dementia, enhance the understanding of the development of dementia, and help to provide better support and services to improve the quality of life of dementia patients and their families.

## Data Availability

The original contributions presented in the study are included in the article/Supplementary material, further inquiries can be directed to the corresponding author.

## Supplementary material

SUPPLEMENTARY FIGURE S1 Receiver operating characteristic (ROC) curve of related indicators for the prediction of dementia in AD patients. **(A)** The area under the curve (AUC) of heart rate acceleration capacity (AC), lipoprotein-associated phospholipase A2 (Lp-PLA2), and AC+Lp-PLA2 for the prediction of dementia in male patients are 0.746, 0.963, and 0.957, respectively. **(B)** The AUC of deceleration capacity (DC), heart rate deceleration 2 (DR2), heart rate deceleration 4 (DR4), heart rate deceleration 8 (DR8), Lp-PLA2 and combination of five variables for the prediction of dementia in male patients are 0.772, 0.891, 0.872, 0.835, 0.963, and 0.972. **(C)** The AUC of AC, Lp-PLA2, and AC+Lp-PLA2 for the prediction of dementia in female patients are 0.764, 0.852, and 0.925. **(D)** The AUC of DC, DR2, DR4, DR8, Lp-PLA2, and combination of five variables for the prediction of dementia in female patients are 0.810, 0.665, 0.876, 0.897, 0.852, and 0.985. The ideal AUC is 1.0. The reference line (black, diagonal line) represents an AUC of 0.5, which is based on chance alone.
