# Supplementary figures and images for: Assessment of heart rate deceleration capacity, heart rate deceleration runs, heart rate acceleration capacity, and lipoprotein-related phospholipase A2 as predictors in individuals with dementia
Source: Front Neurol. 2025 Jan 9;15:1438736. doi: 10.3389/fneur.2024.1438736 (PMC11754063; doi:10.3389/fneur.2024.1438736)

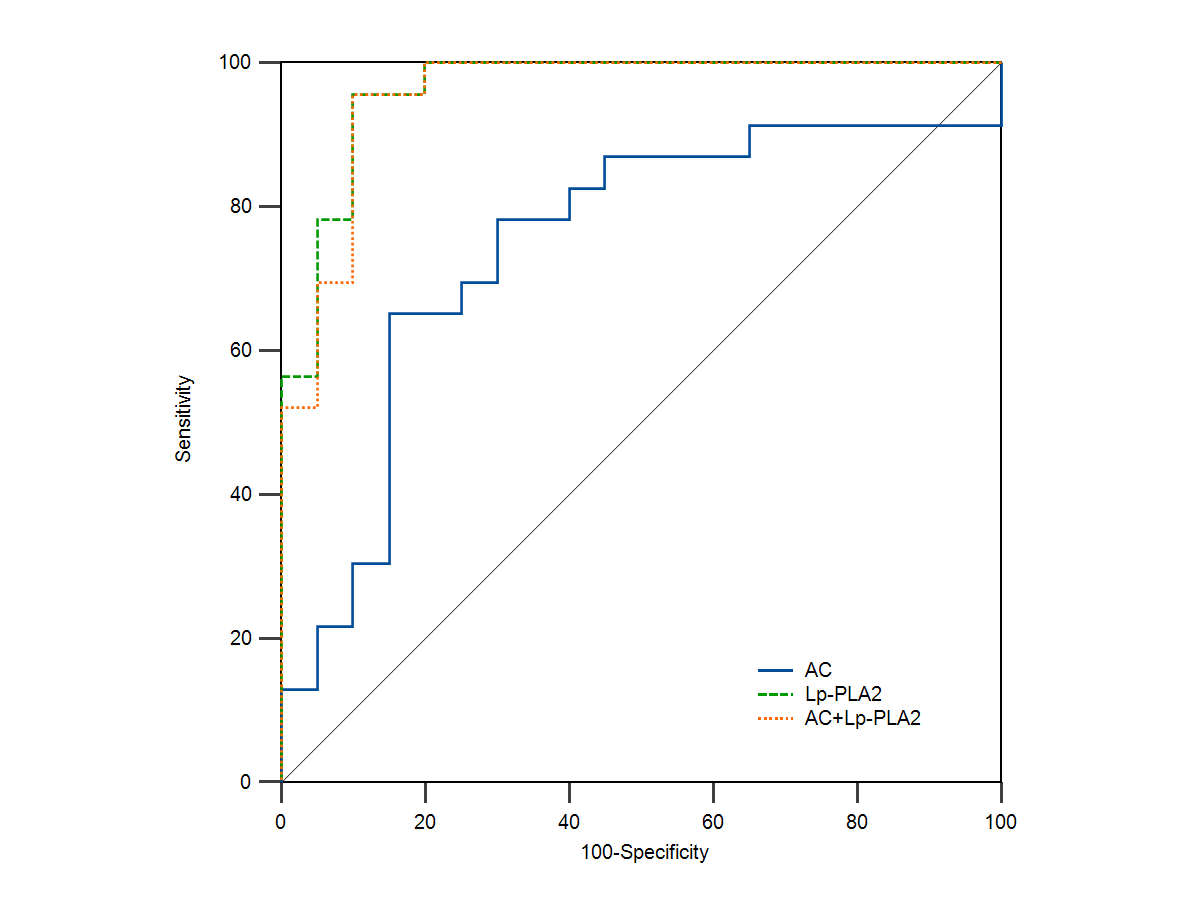

Supplement: SUPPLEMENTARY FIGURE S1 — Receiver operating characteristic (ROC) curve of related indicators for the prediction of dementia in AD patients. (A) The area under the curve (AUC) of heart rate acceleration capacity (AC), lipoprotein-associated phospholipase A2 (Lp-PLA2), and AC+Lp-PLA2 for the prediction of dementia in male patients are 0.746, 0.963, and 0.957, respectively. (B) The AUC of deceleration capacity (DC), heart rate deceleration 2 (DR2), heart rate deceleration 4 (DR4), heart rate deceleration 8 (DR8), Lp-PLA2 and combination of five variables for the prediction of dementia in male patients are 0.772, 0.891, 0.872, 0.835, 0.963, and 0.972. (C) The AUC of AC, Lp-PLA2, and AC+Lp-PLA2 for the prediction of dementia in female patients are 0.764, 0.852, and 0.925. (D) The AUC of DC, DR2, DR4, DR8, Lp-PLA2, and combination of five variables for the prediction of dementia in female patients are 0.810, 0.665, 0.876, 0.897, 0.852, and 0.985. The ideal AUC is 1.0. The reference line (black, diagonal line) represents an AUC of 0.5, which is based on chance alone. [file Image_1.TIF]

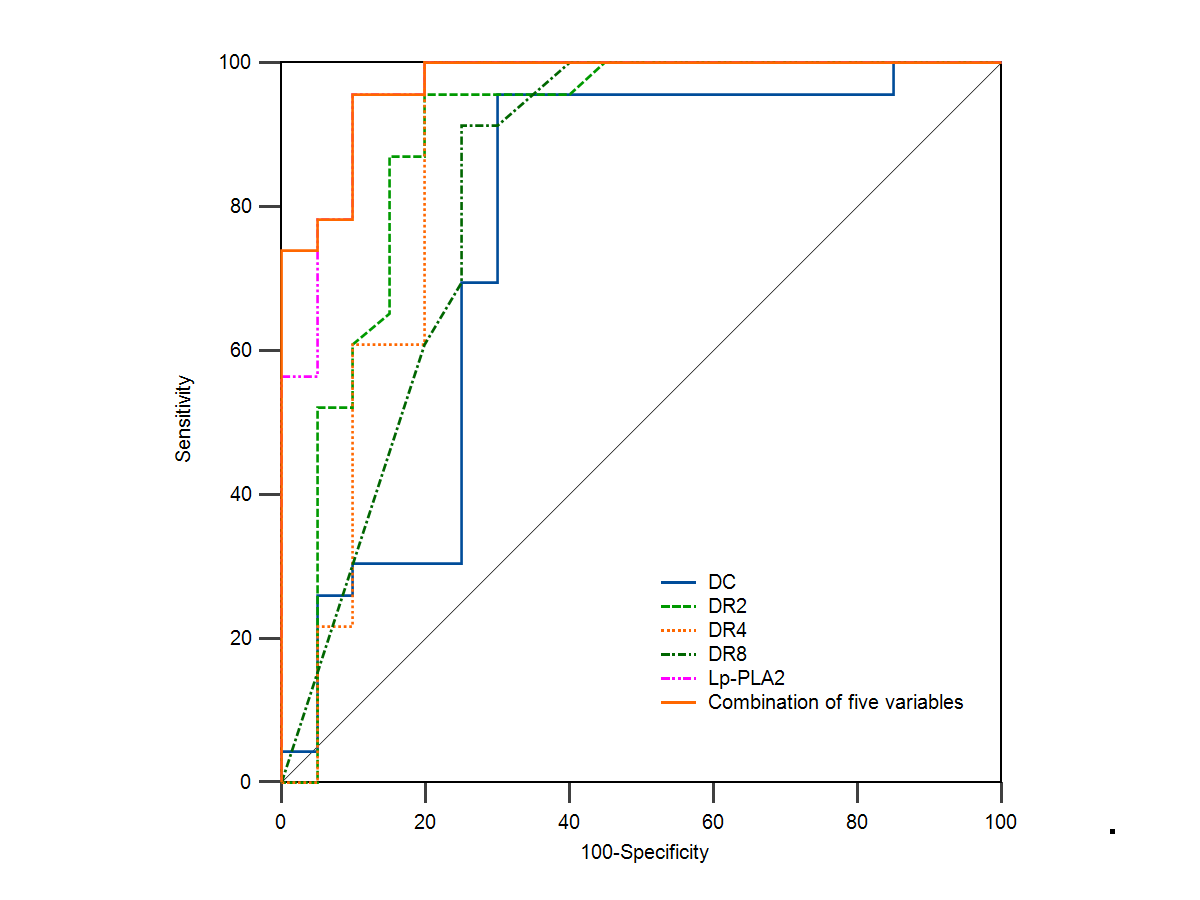

Supplement: Supplementary file 2 [file Image_2.TIF]

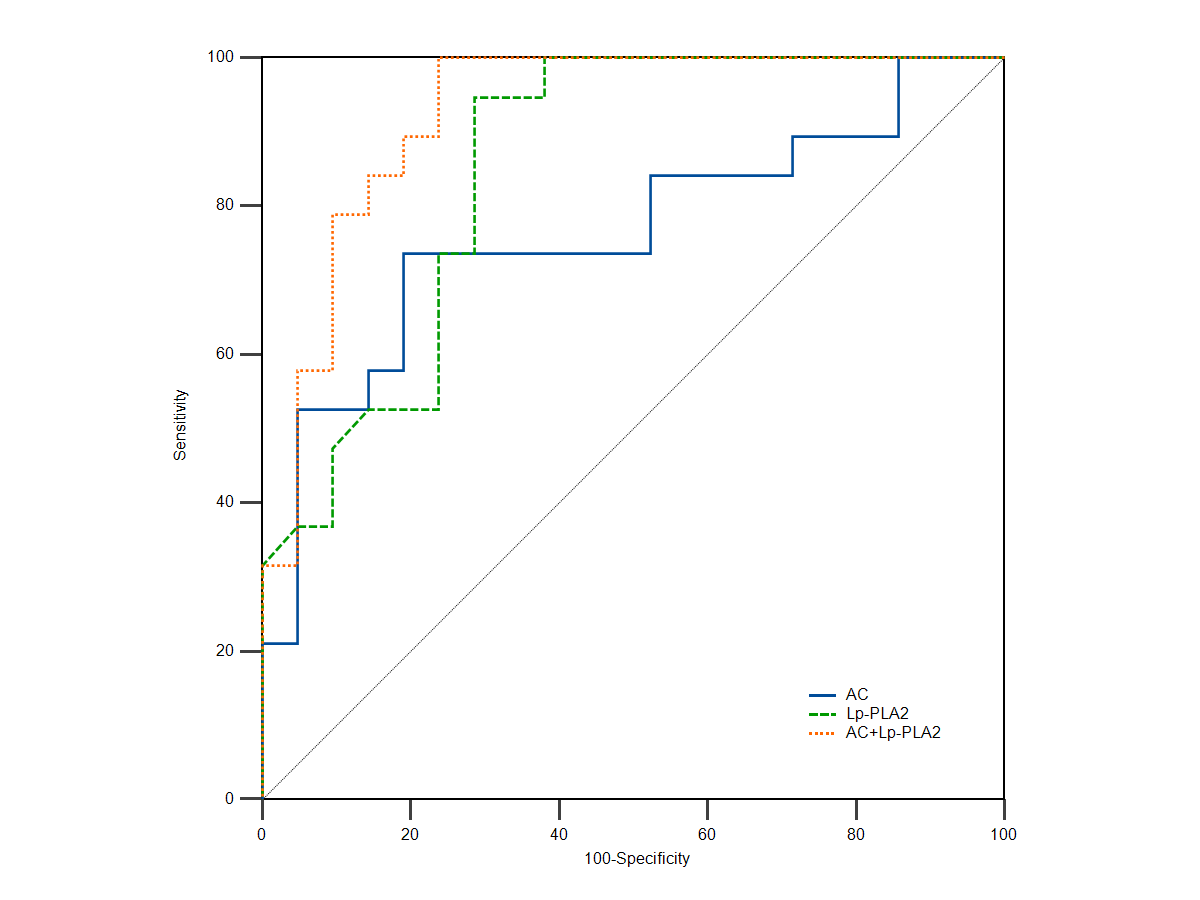

Supplement: Supplementary file 3 [file Image_3.TIF]

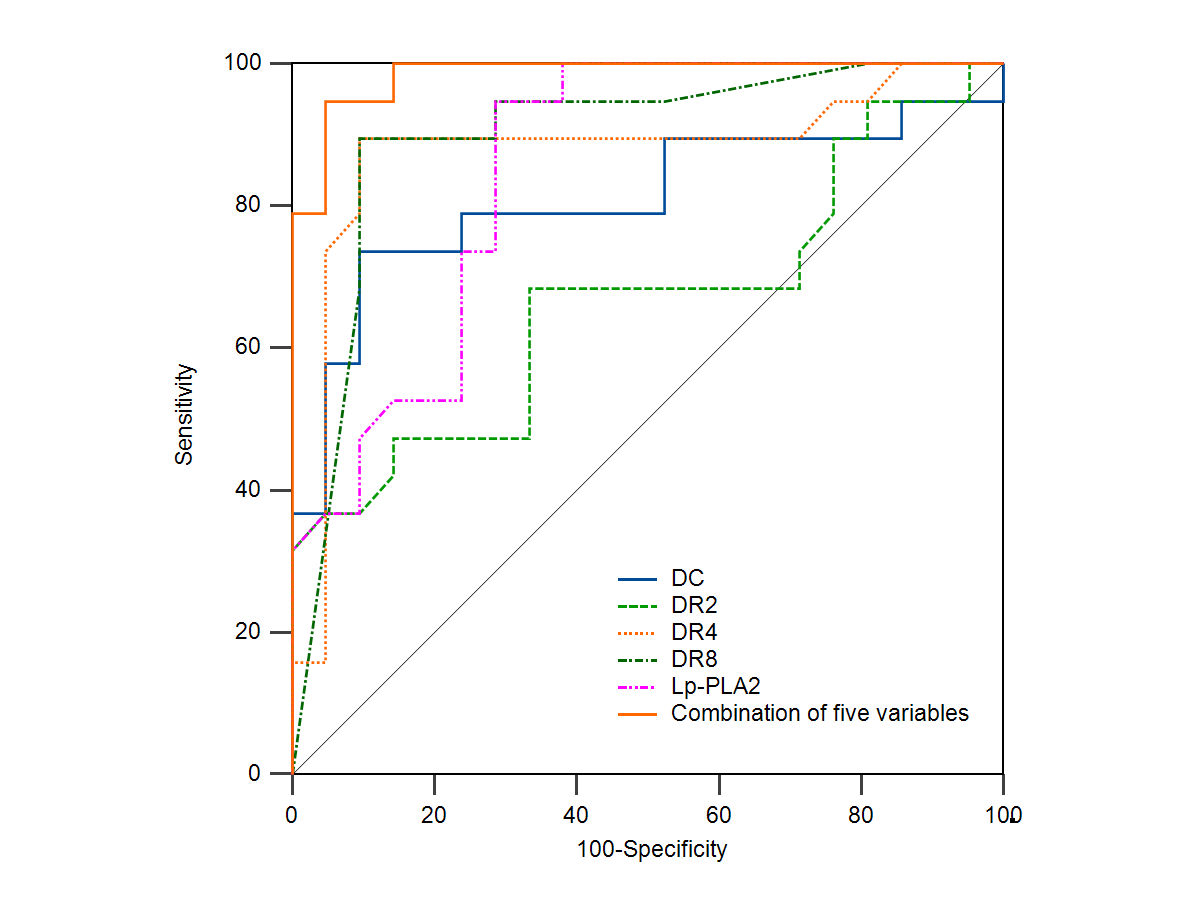

Supplement: Supplementary file 4 [file Image_4.TIF]
